# Jatrorrhizine reduces myocardial infarction-induced apoptosis and fibrosis through inhibiting p53 and TGF-β1/Smad2/3 pathways in mice

**DOI:** 10.1590/acb370705

**Published:** 2022-10-28

**Authors:** Mingxiu Hao, Kunli Jiao

**Affiliations:** 1 MD. Shanghai Jiao Tong University – School of Medicine – Ren Ji Hospital – Department of Geriatrics – Shanghai, China.; 2 MD. Shanghai Jiao Tong University – School of Medicine – Xin Hua Hospital – Department of Cardiology – Shanghai, China.

**Keywords:** Alkaloid, Myocardial Infarction, Apoptosis, Fibrosis, Mice

## Abstract

**Purpose:**

To explore the mechanism of jatrorrhizine on apoptosis and fibrosis induced by myocardial infarction (MI) in an animal model.

**Methods:**

The left anterior descending branch of coronary artery was surgically ligated to duplicate the mouse model of MI. The sham and infarcted mice were treated with normal saline once a day, while mice in experimental groups received low-dose (LD) and high-dose (HD) jatrorrhizine once a day respectively. Two weeks later, cardiac function was detected by echocardiography, and histopathological examination was performed using hematoxylin and eosin (H&E) and Masson staining. The expressions of p53, TGF-β1, Smad/2/3, Bax, Bcl-2, collagen I and collagen III were quantified using qRT-PCR and western blot assays.

**Results:**

Jatrorrhizine significantly improved left ventricular ejection fraction (LVEF) and left ventricle end-systolic (LVES) in mice. Histopathological, administration of jatrorrhizine weakened infiltration of inflammatory cells and cardiac fibrosis in myocardium of mice caused by MI. Additionally, jatrorrhizine suppressed cardiomyocyte apoptosis exhibited as its capability to reverse changes of Bax and Bcl-2 levels in myocardium caused by MI. Jatrorrhizine statistically significantly downregulated expression of collagen I and collagen III, as well as TGF-β1, Smad2/3 and p53.

**Conclusions:**

Jatrorrhizine reduce cardiomyocyte apoptosis and fibrosis through inhibiting p53/Bax/Bcl-2 and TGF-β1/Smad2/3 signaling pathways.

## Introduction

Myocardial infarction (MI) is a common cardiovascular disease with high mortality and disability worldwide^1^. Acute MI leads to cardiomyocyte reduction, scar formation and left ventricular remodeling (LVR), and ventricular remodeling usually results in ventricular dysfunction, eventually heart failure and even death^2^. Clinically, several therapeutic drugs are extensively used, including angiotensin II receptor blockers, angiotensin-converting enzyme (ACE) inhibitors and β-adrenergic blockers, but their efficacy in the prevention and treatment of left ventricular remodeling is limited^3^. Therefore, more effective pharmacological methods need to be developed to improve ventricular remodeling and cardiac function after MI, thereafter to prevent heart failure.

Cardiomyocyte apoptosis occurs after MI, which dominantly due to long-term ischemia^4^. Thus, preventing cardiomyocyte from apoptosis is an effective way to significantly improve left ventricular remodeling and chronic heart failure after MI^5^. Increasing evidences have documented that p53 is one of the most critical molecules mediating apoptosis through its capability to regulate downstream molecules, especially up-regulate the expression of Bax and down-regulate the expression of Bcl-2^6^^,^ 7. Theoretically, inhibiting p53 signaling pathway may benefit to suppress apoptosis and thus prevent MI. On the other hand, cardiomyocyte apoptosis usually leads to cardiac fibrosis characterized as myofibroblast formation and excessive secretion of extracellular matrix^8^, which could eventually result in heart failure^9^. Being a pivotal regulator of cardiac fibrosis, transforming growth factor-β1 (TGF-β1) binds to TGFβ receptor on plasma membrane and thus mediates phosphorylation of Smad2/3. Then, phosphorylated Smad2/3 interacts with Smad4 to form complex, which translocate into nucleus and initiate gene transcription^10^. Stimulation of TGF-β1 leads to expression of various downstream genes, and eventually result in activation of fibroblasts, and therefore promotes the production of extracellular matrix^11^. Preclinical study showed that TGF-β1 was excessively expressed in myocardium after MI, and blockage of TGF-β1 signaling pathway statistically reduced cardiac fibrosis induced by MI^12^. Hence, targeting TGF-β1/Smad signaling pathway may be a potential strategy of MI treating.

Jatrorrhizine is a natural protoberberine alkaloid, and has antidotal, sterile, hypoglycemic and hypolipidemic bioactivity^13^. Previous experimental studies have proved that jatrorrhizine has a protective effect on myocardial ischemia and reperfusion injury^14^. However, the efficacy of jatrorrhizine on cardiac function after MI and its potential mechanism is still unknown.

In this study, MI model was duplicated in mouse, and the effect of jatrorrhizine on MI induced mice, as well as the potential protective mechanism of jatrorrhizine on MI, was investigated. This will lay the foundation for the treatment of MI for the time to come.

## Methods

Isoflurane was purchased from Shenzhen RWD Life Science Co., Ltd; Primary antibodies against TGF-1β (3711S), Smad2/3 (8685s) were purchased from Cell Signaling Technology Inc (Beverly, Ma, USA). Primary antibodies against Bcl-2 (Cat No. 12789-1-AP) and p53 (Cat No. 10442-1-AP) were purchased from Proteintech (Chicago, USA). Primary antibodies against Bax (Cat No. AB7977) were purchased from Abcam (Cambridge Science Park, England). Primary anti-β-actin (cat No. SAB3500350) antibody was purchased from Abbkine (California, United States). Jatrorrhizine was purchased from Dalian Meilun Biotech Co., Ltd. Before experiment, jatrorrhizine was dissolved in corresponding amount of saline at room temperature.

### Experimental animals

Male C57BL/6 mice (10–12 weeks old; 20–22g) purchased from Shanghai SLAC Experimental Animal Co., Ltd. (Shanghai, China) were housed at room temperature (24 ± 2 °C) with 50–60% humidity under 12–12 light/dark cycles. Before experimental study, mice were adaptively fed for 3 days. The animal experiments performed in this study was approved by the animal ethics committee of Xin Hua Hospital, School of Medicine, Shanghai Jiao Tong University (approval No. SHRM-IACUC-044), and carried out in accordance with the guidelines for the conservation and utilization of experimental animals and the principles for the conservation and utilization of vertebrates.

### Animal model of MI and administration of jatrorrhizine

MI model was duplicated by ligation of left anterior descending branch of coronary artery using 6-0 silk suture. Echocardiography was performed to assess mice with MI, which was confirmed by observing the blood flow of left ventricle. The ejection fraction of the mice was approximately 35% ± 10% before drug intervention. After MI incubation, mice were randomly divided into three groups: Myocardial infarction group treated with intragastric normal saline (MI, n = 6); Myocardial infarction plus low-dose (2.5 mg/kg/day) (LD, n = 6) and high-dose jatrorrhizine (5 mg/kg/day) (HD, n = 6) treatment group. Sham operation group received intragastric normal saline (NC, n = 6); The sham mice underwent the same operation without ligation. Twenty-four hours after operation, mice in the sham group and MI group were intragastric administrated with normal saline, and mice in the jatrorrhizine group received LD and HD of jatrorrhizine once a day for 14 days. Finally, mice were used for cardiac echocardiography, and then sacrificed for subsequent experiments.

### Cardiac echocardiography

Transthoracic echocardiography was performed after 14 days using Vevo 2100 imaging system outfitted with a L12-5 linear broadband and an S12 phased array transducer equipped with a 0.3 cm standoff. After been anesthetized with isoflurane, mice were lay on their back on a heating platform, and heart rate was maintained 400–450 per minute. The images were obtained in two-dimensional mode from parasternal long axis view. Left ventricular ejection fraction (LVEF) and left ventricle end-systolic (LVES) were recorded. Image analysis was performed using VEVO Strain software.

### Histopathological assessment

The hematoxylin and eosin (H&E) and Masson staining were performed as previously described^15^. In brief, the heart sample was fixed in 4% paraformaldehyde, embedded in paraffin, and cut into 4 μm-thick cross-sections. Then the paraffin sections were prepared and submitted to H&E staining (hematoxylin, 37 °C for 3–5 min; eosin, 37 °C for 5–15 s). The histopathological changes were observed by H&E staining. In addition, Masson trichrome staining (37 °C for 1–5 min) was also performed to identify collagen deposition and infarct size. The slices were then examined by optical microscopy and the images were captured at 400 times magnification.

### Quantitative real-time polymerase chain reaction (QRT-PCR)

The changes of mRNA levels of Bax, Bcl-2, collagen I and collagen III were analyzed in this study by using qRT-PCR. In brief, total RNAs of whole hearts were extracted and then reverse transcribed according to the manufactures’ instructions. Amplification was quantified with SYBR premix Ex Taq2. Gene expressions were normalized to β-actin level and analyzed by 2^-ΔΔCt^ method. The primers used were as follows: Bax, F: 5’- TGGCAGCTGACATGTTTTCTGAC-3’, R: 5’- TCACCCAACCACCCTGGTCTT-3’; Bcl2, F: 5’-AGAACCTTGTGTGACAAATGAGAAC-3’, R: 5’-TACCCATTAGACATATCCAGCTTGA-3’; Collagen I, F: 5’-CCCCCTCCCCAGCCACAAAG-3’, R: 5’-TCTTGGTCGGTGGGTGACTCT-3’; Collagen III, F: 5’-CCAAACTCTATCTGAA-3’, R: 5’-GGACTCATAGAATACA-3’; β-actin, F: 5’- ATCTGGCACCACACCTTCTA-3’, R: 5’- CGTCATACTCCTGCTTGCTG-3’.

### Western blot

The whole hearts were cut into small pieces, then lysed on ice with cell lysis buffer supplemented with protease inhibitor and PMSF for 30 min. Then, samples were centrifuged at 12,000 g at 4 °C for 15 min. The concentration of total protein was determined using BCA protein detection kit (Thermo Scientific, Waltham, MA, USA); 50 μg total protein was separated on 10% SDS-PAGE gel, and then transferred to 0.45 μm PVDF membrane (Amersham Hybond, GE Healthcare, München, Germany). The PVDF membrane was blocked with TBST (Tris buffered saline, 0.2% Tween) buffer and 5% milk at room temperature for 2 h, and then incubated at 4 °C overnight with primary antibodies: rabbit anti-TGF-β1 (1:1,000), rabbit anti-Smad2/3 (1:1,000), rabbit anti-p53 (1:1,000), rabbit anti-Bax (1:1,000), rabbit anti-Bcl-2 (1:1,000), rabbit anti-β-actin (1:5,000). After been washed with TBST buffer, the membrane was incubated with goat anti-rabbit secondary antibody at room temperature for 1 h. Subsequently, the membrane was washed with TBST buffer and tested with ECL Kit (ThermoFisher, USA). Protein expression of β-actin was used as an internal control. Image J software (National Institutes of Health, Bethesda, Maryland, USA) was used for gray-scale quantitative analysis.

### Statistical analysis

The data were expressed as mean ± SD using SPSS 20.0. Differences between two groups were calculated with the one-way analysis of variance (ANOVA), followed by Dunnett’s post-hoc test. For the data that do not conform to the normal distribution, the nonparametric Kruskal–Wallis test was used to compare the differences between the three groups. P < 0.05 was considered as significant threshold.

## Results

### Jatrorrhizine improved cardiac function in mice with MI

**Figure 1:**
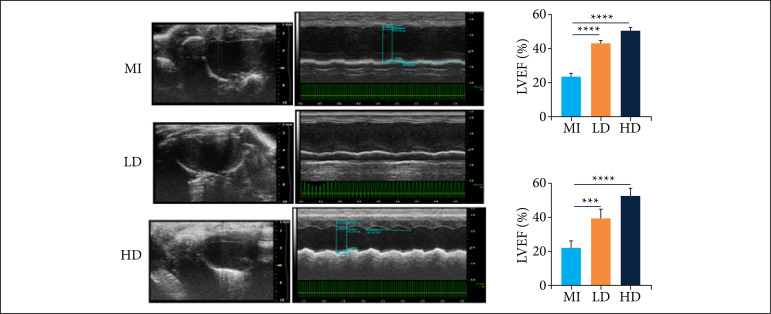
Effect of jatrorrhizine on LVEF and LVES of mice with MI.

MI: MI mice received normal saline; LD: MI mice received low dose jatrorrhizine; HD: MI mice received high dose jatrorrhizine. ^***^ p < 0.001, ^****^ p < 0.0001.

Two weeks after ligation of the left anterior descending branch of coronary artery, the cardiac function of mice was analyzed by echocardiography. As shown in Fig. 1, significantly reduced LVEF and LVES were detected in mice with MI. After been treated with jatrorrhizine, both LVEF and LVES were statistically increased. These findings indicated that jatrorrhizine should significantly inhibit the decline of cardiac function induced by MI in mice.

### Jatrorrhizine improved myocardial histopathological changes in mice with MI

**Figure 2:**
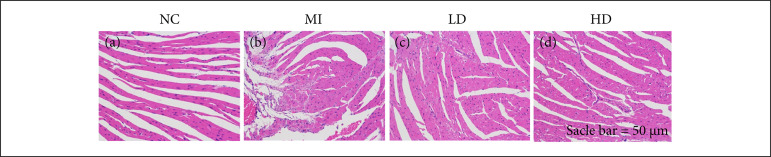
Histopathological examination of effect of jatrorrhizine in mice with MI. a-d: H&E staining. Intact structure of myocardium **(a)** was lost due to MI, instead of massive fibrosis and infiltration of inflammatory cells **(b)**; administration of LD jatrorrhizine **(c)** and HD jatrorrhizine **(d)** effectively recovered myocardial structure, and significantly weaken fibrosis and infiltration of inflammatory cells.

NC: sham-operated mice received normal saline; MI: MI mice received normal saline; LD: MI mice received low dose jatrorrhizine;HD: MI mice received high dose jatrorrhizine. Scale bar = 50 μm, magnification 100×.

**Figure 3:**
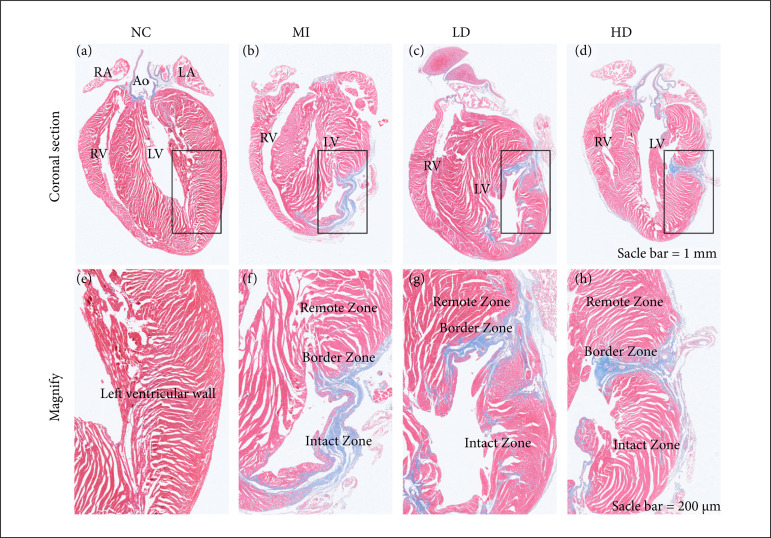
Representative pictures for infarct by Masson staining in mice heart. **(a-d)** Masson staining in the coronal section of mice heart. Scale bar: 1 mm, magnification 1.25×. The black box area is enlarged in the lower panel; **(e-h)** Enlarged view of the black box area in a-d, respectively. Scale bar: 200 μm, magnification 5×. After administration of jatrorrhizine, the infarct area is significantly decreased.

The cardioprotective effect of jatrorrhizine was evaluated histopathologically here by using H&E and Masson staining. As shown in Fig. 2, mice in NC control group displayed intact myocardium, and fibrosis was undetectable. MI incubation resulted in loss of intact myocardium, severe infiltration of inflammatory cells and massive fibrosis in myocardium. However, pathological changes above mentioned were statistically weakened after been administered with jatrorrhizine, instead of recovered myocardial structure, fewer fibrosis and infiltration of inflammatory cells. Moreover, MI treatment caused the obvious infarct in mice heart compared with that in NC group (Fig. 3a, b and Fig. 3e, f); while the infarct area was significantly decreased after the administration of jatrorrhizine (Fig. 3c, d and Fig. 3g, h). These data suggested that jatrorrhizine effectively weakens of MI induced injury in mice.

RV: right ventricular. LV: left ventricular. NC: sham-operated mice received normal saline; MI: MI mice received normal saline; LD: MI mice received low dose jatrorrhizine; HD: MI mice received high dose jatrorrhizine.

### Jatrorrhizine attenuated cardiomyocyte apoptosis and cardiac fibrosis induced by MI

**Figure 4:**
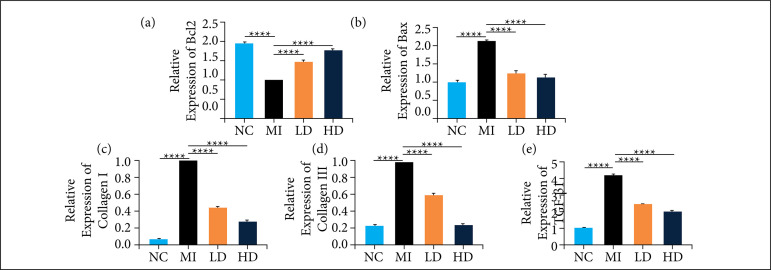
Effect of jatrorrhizine on the expression of apoptosis and cardiac fibrosis-related proteins in mice. MI generated statistical downregulation in Bcl-2 mRNA and upregulation in Bax mRNA level, and these changes were partly recovered after been administration of jatrorrhizine **(a, b)**. MI resulted in statistical upregulation of collagen I, collagen III and TGF-β1, which as significantly reduced by jatrorrhizine. NC: sham-operated mice received normal saline **(c-e)**.

MI: MI mice received normal saline; LD: MI mice received low dose jatrorrhizine; HD: MI mice received high dose jatrorrhizine. ^****^ p < 0.0001.

To explore whether jatrorrhizine suppress infarction induced apoptosis, expressions of Bax and Bcl-2 in mRNA level were detected. As shown in Fig. 4a and b compared to those in NC group, statistical down-regulation of Bcl-2 and up-regulation of Bax were detected in infarcted myocardium of mice. But, changes of Bax and Bcl-2 were partly reversed after been treated with jatrorrhizine, exhibited as statistical upregulation of Bcl-2 and downregulation of Bax.

In order to identify further whether jatrorrhizine enable to reduce cardiac fibrosis caused by MI, the mRNA levels of collagen I and collagen III in myocardium of each experimental mice were detected. As shown in Fig. 4c and d, expressions of collagen I and collagen III were lower in sham-operated mice. However, both collage I and III were statistically up-regulated after myocardium been infarcted. Aberrant overexpression of either collagen I and collagen III in infarcted myocardium was significantly suppressed due to administration of jatrorrhizine. Especially, level of collagen III in animals treated with high dose jatrorrhizine was comparable to those in normal control group. In addition, the expression of TGF-β1 was significantly upregulated in infarcted myocardium of mice, while jatrorrhizine treatment obviously reduced its expression (Fig. 4e). These findings indicated that jatrorrhizine enable to weaken cardiac fibrosis caused by MI.

### Jatrorrhizine reduced infarction induced cardiac fibrosis through inhibiting TGF-β1/Smad2/3 and p53 signaling pathway

**Figure 5:**
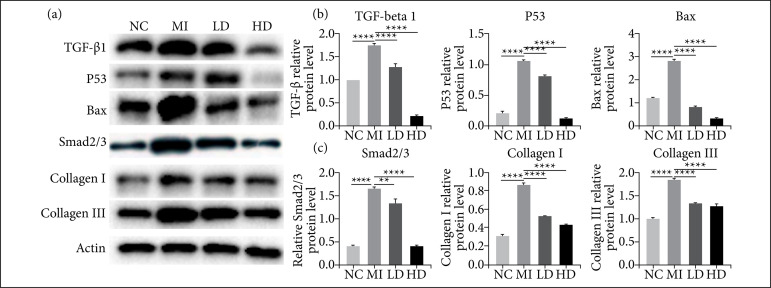
Role of jatrorrhizine on expressions of TGF-β1/Smad2/3 and p53 in mice. MI led tostatistical upregulation in TGF-β1, Smad2/3 and p53 protein levels, which were significantlyreduced by administration of jatrorrhizine. Western blot **(a)** and statistic analysis **(b)**.

NC: sham-operated mice received normal saline; MI: MI mice received normal saline; LD: MI mice received low dose jatrorrhizine;HD: MI mice received high dose jatrorrhizine. ^**^ p < 0.01, ^****^ p < 0.0001.

It has been documented that Smad signaling pathway plays important role to mediate development of cardiac fibrosis^16^. Thus, expression changes of TGF-β1 and Smad2/3, as well as p53 were detected here by western blot. Data showed that MI led to statistical upregulation of TGF-β1, Smad2/3 and p53 in myocardium, which was statistically decreased after been treated with jatrorrhizine. Interestingly, administration of high dose jatrorrhizine showed more reduced levels of TGF-β1 and Smad2/3 that comparable to those in normal control (Fig. 5a, b). Meanwhile, the expression of Bax, collagen I and collagen III was consistent to that detected by PCR. Taken together, data abovementioned suggested that jatrorrhizine enable to reduce cardiac fibrosis through inhibiting TGF-β1/ Smad2/3 and p53 signaling pathway.

## Discussion

Data present in this study demonstrated that administration of jatrorrhizine enabled significantly improvement of cardiac function and reduced cardiomyocyte apoptosis and fibrosis in a mouse model of MI. Jatrorrhizine alleviated MI induced cardiomyocyte apoptosis through inhibiting p53 signaling pathway, and reversed myocardial fibrosis by inhibiting TGF-β1/Smad2/3 signaling pathway. Based on the findings, jatrorrhizine could be used in clinic to treat MI.

MI is still clinically challenge, partly due to limited treatment. So, developing novel treatment is an urgent problem to be solved. Currently, Chinese herbs have attracted extensive attention because they contain variety of natural compounds therefore to prevent and treat various diseases through various approaches. Jatrorrhizine is an alkaloid in *Coptis chinensis*, and has been experimentally proven to have a protective effect on myocardial ischemia and reperfusion injury^13^. However, the therapeutic effect and mechanism of jatrorrhizine on coronary heart disease are still unclear. This research indicated that jatrorrhizine treatment enabled to significantly improve the LVEF and LVES, and partly recovered myocardial structure in mice with MI. Hence, potential molecular mechanism mediating cardioprotective effect of jatrorrhizine was explored here in order to provide basis for its clinical application.

Various molecules and signaling pathways participate in initiation and development of MI, including HIF-1α, ROS and NADPH oxidase, as well as PI3K/Akt and MAPK signals^17^–^19^. Present data showed that administration of jatrorrhizine enabled to reverse expression changes of Bax and Bcl-2 in infarcted myocardium, indicating that jatrorrhizine have potential to suppress cardiomyocyte apoptosis caused by MI. It is well known that p53 is a crucial apoptosis regulator. Therefore, jatrorrhizine may suppress apoptosis through p53 signaling pathway. This experiment showed that MI produced severe cardiomyocyte apoptosis accompanied with overexpression of p53 protein in myocardium. Administration of jatrorrhizine, however, statistically weakened cardiomyocyte apoptosis and down-regulated p53 level. In further, jatrorrhizine exhibited it capability to upregulate mitochondria Bcl-2 and downregulate Bax protein. Taken together, jatrorrhizine could protect cardiomyocyte from apoptosis induced by MI through inhibiting p53 signaling pathway.

According to the theory of traditional Chinese medicine, the main cause of heart failure after MI is Qi deficiency and prolongation of blood stasis, resulting in weakened heart unable to transport blood^20^. Here, pathological examination confirmed that MI generated severe collagen deposition and interstitial fibrosis, which was accordance the previous study^21^. TGF-β1 has been considered to be an important regulator of cardiac fibrosis and plays a pivotal role in ventricular remodeling and heart failure^22^–^24^. Thus, expression changes of key molecules in TGF signaling pathway were quantified in this study. Data revealed that MI resulted in overexpression of TGF-β1 and Smad2/3 in myocardium, and the overexpression of TGF-β1 and Smad2/3 was decreased significantly after mice been treated with jatrorrhizine. These results indicated that administration of jatrorrhizine could enable to weaken infarction induced cardiac fibrosis through inhibiting TGF-β1/Smad2/3 signaling pathway, which should be benefit to prevent collagen deposition. To confirm the findings in this study, the subsequent experiments by expanding the sample size, and also the more specific molecular mechanisms should be performed and investigated in the future.

## Conclusion

Jatrorrhizine has a considerable effect to prevent cardiac remodeling after MI, which attributes to its capability to suppress apoptosis through inhibiting p53/Bax/Bcl-2 signaling pathway and weaken cardiac fibrosis through inhibiting TGF-β1/Smad2/3 signaling pathway. The present study provided an experimental basis for clinic usage of jatrorrhizine to treat MI.

## References

[1] Rastogi A, Novak E, Platts AE, Mann DL (2017). Epidemiology, pathophysiology and clinical outcomes for heart failure patients with a mid-range ejection fraction. Eur J Heart Fail.

[2] Hung J, Teng TH, Finn J, Knuiman M, Briffa T, Stewart S, Sanfilippo FM, Ridout S, Hobbs M. (2013). Trends from 1996 to 2007 in incidence and mortality outcomes of heart failure after acute myocardial infarction: A population-based study of 20,812 patients with first acute myocardial infarction in Western Australia. J Am Heart Assoc.

[3] Horiuchi Y, Tanimoto S, Aoki J, Nakajima H, Hara K, Tanabe K. (2016). Effects of β-blockers on left ventricular remodeling in patients with preserved ejection fraction after acute myocardial infarction. Int J Cardiol.

[4] Teiger E, Than VD, Richard L, Wisnewsky C, Tea BS, Gaboury L, Tremblay J, Schwartz K, Hamet P. (1996). Apoptosis in pressure overload-induced heart hypertrophy in the rat. J Clin Invest.

[5] Hayakawa K, Takemura G, Kanoh M, Li Y, Koda M, Kawase Y, Maruyama R, Okada H, Minatoguchi S, Fujiwara T, Fujiwara H. (2003). Inhibition of granulation tissue cell apoptosis during the subacute stage of myocardial infarction improves cardiac remodeling and dysfunction at the chronic stage. Circulation.

[6] Feng X, Liu X, Zhang W, Xiao W. (2011). p53 directly suppresses BNIP3 expression to protect against hypoxia-induced cell death. Embo J..

[7] Chi SW (2014). Structural insights into the transcription-independent apoptotic pathway of p53. BMB Rep.

[8] Burchfield JS, Xie M, Hill JA. (2013). Pathological ventricular remodeling: Mechanisms: Part 1 of 2. Circulation.

[9] Bacmeister L, Schwarzl M, Warnke S, Stoffers B, Blankenberg S, Westermann D, Lindner D. (2019). Inflammation and fibrosis in murine models of heart failure. Basic Res Cardiol.

[10] Derynck R, Zhang YE (2003). Smad-dependent and Smad-independent pathways in TGF-beta family signalling. Nature.

[11] Dobaczewski M, Chen W, Frangogiannis NG. (2011). Transforming growth factor (TGF)-β signaling in cardiac remodeling. J Mol Cell Cardiol.

[12] Ikeuchi M, Tsutsui H, Shiomi T, Matsusaka H, Matsushima S, Wen J, Kubota T, Takeshita A. (2004). Inhibition of TGF-beta signaling exacerbates early cardiac dysfunction but prevents late remodeling after infarction. Cardiovasc Res.

[13] Zhu SL, Lei T, Gao X, Tu J. (2018). Jatrorrhizine regulates GLUTs with multiple manners for hypoglycemic effect in insulin-resistance 3T3-L1 adipocytes. Zhongguo Zhong Yao Za Zhi.

[14] Tan HL, Chan KG, Pusparajah P, Duangjai A, Saokaew S, Mehmood Khan, Lee LH, Goh BH (2016). Rhizoma Coptidis: A potential cardiovascular protective agent. Front Pharmacol.

[15] Sadayappan S, Gulick J, Osinska H, Martin LA, Hahn HS, Dorn GW, Klevitsky R, Seidman CE, Seidman JG, Robbins J (2005). Cardiac myosin-binding protein-C phosphorylation and cardiac function. Circ Res.

[16] Li X, Zhang ZL, Wang HF. (2017). Fusaric acid (FA) protects heart failure induced by isoproterenol (ISP) in mice through fibrosis prevention via TGF-β1/SMADs and PI3K/AKT signaling pathways. Biomed Pharmacother.

[17] Mizuno M, Kuno A, Yano T, Miki T, Oshima H, Sato T, Nakata K, Kimura Y, Tanno M, Miura T. (2018). Empagliflozin normalizes the size and number of mitochondria and prevents reduction in mitochondrial size after myocardial infarction in diabetic hearts. Physiol Rep.

[18] Zhang XG, Wei Y, Jiang J, Wang L, Liang HY, Lei CB (2020). Effect of TGF-β1 on myocardial cell apoptosis in rats with acute myocardial infarction via MAPK signaling pathway. Eur Rev Med Pharmacol Sci.

[19] Zhang Q, Lu L, Liang T, Liu M, Wang ZL, Zhang PY (2017). MAPK pathway regulated the cardiomyocyte apoptosis in mice with post-infarction heart failure. Bratisl Lek Listy.

[20] Hu G, Yang P, Zeng Y, Zhang S, Song J. (2018). Danggui Buxue decoction promotes angiogenesis by up-regulation of VEGFR(1/2) expressions and down-regulation of sVEGFR(1/2) expression in myocardial infarction rat. J Chin Med Assoc.

[21] Zhang HQ, Yau YF, Wong MS, Man OY, He YY, Chan N, Li M. (2008). Chinese medicine formula DSQRL versus glucocorticoids for the treatment of experimental pulmonary fibrosis. J Ethnopharmacol.

[22] Hu HH, Chen DQ, Wang YN, Feng YL, Cao G, Vaziri ND, Zhao YY. (2018). New insights into TGF-β/Smad signaling in tissue fibrosis. Chem Biol Interact.

[23] Hojo Y, Saito T, Kondo H. (2012). Role of apoptosis in left ventricular remodeling after acute myocardial infarction. J Cardiol.

[24] Karetnikova VN, Kashtalap VV, Kosareva SN, Barbarash OL (2017). Myocardial fibrosis: Current aspects of the problem. Ter Arkh.

