# ERRATA

**DOI:** 10.1590/acb370705.errata

**Published:** 2023-03-13

In the manuscript **Jatrorrhizine reduces myocardial infarction-induced apoptosis and fibrosis through inhibiting p53 and TGF-β1/Smad2/3 pathways in mice**, which DOI is: https://doi.org/10.1590/acb370705, published in the journal **Acta Cirúrgica Brasileira**, 2022;37(07)e370705, page 1, in affiliation:

**Instead of**:

2. MD. Shanghai Jiao Tong University – School of Medicine – Ren Ji Hospital – Department of Cardiology – Shanghai, China.

**Should be**:

2. MD. Shanghai Jiao Tong University – School of Medicine – Xin Hua Hospital – Department of Cardiology - Shanghai, China.

